# STM Visualization of N_2_ Dissociative Chemisorption on Ru(0001) at High Impinging Kinetic Energies

**DOI:** 10.1021/acs.jpcc.2c05770

**Published:** 2022-10-19

**Authors:** Joshua Wagner, Tim Grabnic, S. J. Sibener

**Affiliations:** The James Franck Institute and Department of Chemistry, The University of Chicago, 929 East 57th Street, Chicago, Illinois 60637, United States

## Abstract

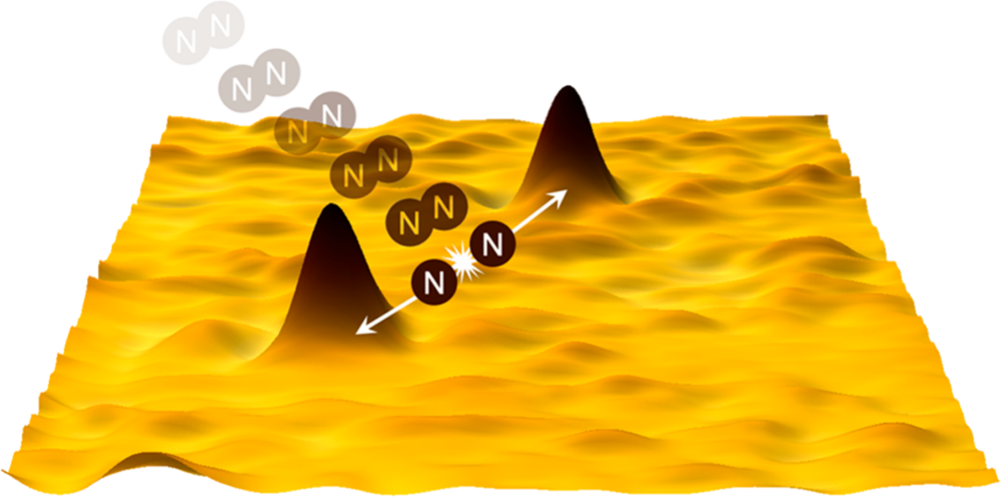

This paper examines the reactive surface dynamics of energy- and angle-selected N_2_ dissociation on a clean Ru(0001) surface. Presented herein are the first STM images of highly energetic N_2_ dissociation on terrace sites utilizing a novel UHV instrument that combines a supersonic molecular beam with an *in situ* STM that is in-line with the molecular beam. Atomically resolved visualization of individual N_2_ dissociation events elucidates the fundamental reactive dynamics of the N_2_/Ru(0001) system by providing a detailed understanding of the on-surface dissociation dynamics: the distance and angle between nitrogen atoms from the same dissociated N_2_ molecule, site specificity and coordination of binding on terrace sites, and the local evolution of surrounding nanoscopic areas. These properties are precisely measured over a range of impinging N_2_ kinetic energies and angles, revealing previously unattainable information about the energy dissipation channels that govern the reactivity of the system. The experimental results presented in this paper provide insight into the fundamental N_2_ dissociation mechanism that, in conjunction with ongoing theoretical modeling, will help determine the role of dynamical processes such as energy transfer to surface phonons and nonadiabatic excitation of electron–hole pairs (ehps). These results will not only help uncover the underlying chemistry and physics that give rise to the unique behavior of this activated dissociative chemisorption system but also represent an exciting approach to studying reaction dynamics by pairing the angstrom-level spatiotemporal resolution of an *in situ* STM with nonequilibrium fluxes of reactive gases generated in a supersonic molecular beam to access highly activated chemical dynamics and observe the results of individual reaction events.

## Introduction

The interaction of gaseous species on ruthenium surfaces has been studied extensively due to the importance of ruthenium as a catalyst for a wide variety of applications.^1,2^ Notably, Ru-based systems represent an important class of potential second-generation catalysts for synthetic ammonia production through the Haber–Bosch process due to the higher activity under milder conditions of ruthenium as compared to iron catalysts.^3−10^ The rate-limiting step of this process is the dissociative chemisorption of N_2_^11^ due to the high amounts of energy required to break its strong triple bond. A fundamental understanding of N_2_ dissociation onto ruthenium surfaces is therefore of great fundamental, technological, and economic importance.^12^

In addition to its relevance to ammonia synthesis, N_2_ dissociation onto Ru(0001) is a prototypical activated dissociative chemisorption process, and understanding the mechanistic features of this would have a considerable impact on the field of heterogeneous catalysis. Compared to other activated dissociation benchmark systems—namely H_2_ on Cu^13−15^ or CH_4_ on transition metals—that have dissociation probabilities (*s*_0_) that approach unity at normal incident kinetic energies (*E*_*N*_) much greater than the potential barrier (V*),^16−18^ N_2_ exhibits substantially different adsorption behavior, demonstrating *s*_0_ ≪ 1 at *E*_*N*_ ≫ V*.^19−21^ Nonadiabatic coupling/tunneling mechanisms^19,22−28^ and energy transfer to surface phonons,^29,30^ along with theoretical formulations using only adiabatic treatments,^31−33^ have all been proposed to describe the unusual dissociation behavior of N_2_ on Ru(0001) with increasing number of degrees of freedom explicitly treated in forming the potential energy surface—some formulations treating several degrees^19,22,23,25^ and some formulations treating all six degrees of freedom for the nitrogen molecule.^32−34,34−36^ Neural networks have been used to treat all degrees of freedom explicitly using a high-dimensional fit of molecule–surface interactions, allowing for less computationally expensive ab initio molecular dynamics simulations for a system in which nonreactive scattering dominates (*s*_0_ ≪ 1), making reactivity more arduous to sample.^26,29,30^ Neural networks have also been used to sample the free energy surface of nitrogen dissociation on Ru(0001) showing vibrational entropy of surface atoms add appreciably to the reaction barrier.^37^ Further experimental work is required to answer questions that remain about the fundamental reactive surface dynamics of this important system.

In this paper, we present results detailing the reactive dynamics of N_2_ on a clean Ru(0001) surface held at room temperature and 262 K. Atomically resolved visualization of individual dissociation events at different impinging energies and angles provide a detailed understanding of the spacing and angle of nitrogen atoms from the same dissociated N_2_ molecule, site specificity of terrace binding, and local, nanoscopic information about the reactive evolution of the Ru(0001) surface in the low-coverage regime. By monitoring the nanoscopic evolution of the Ru(0001) surface during exposure to energy- and angle-selected N_2_, this work elucidates new information about the mechanisms of energy dissipation into the surface of this important gas–surface interface and more generally showcases how nonequilibrium fluxes of reactant molecules from a supersonic molecular beam paired with an in-line *insitu* STM can capture the fundamental dynamics of individual reaction events. In conjunction with ongoing and future theoretical studies, observation of individual dissociation events will reveal insight into how energy is dissipated into the surface and may divulge the role surface phonons and nonadiabatic coupling to electron–hole pairs (ehps) play in transferring energy during and immediately after the activated dissociation event.

The dissociation probability of N_2_ on a clean Ru(0001) surface under ambient conditions is very low (*s*_0_ ≈ 10^–12^)^38−40^ due to a high activation barrier that occurs late in the dissociation process and requires significant stretching of the N_2_ bond.^20,24,33,41^ Thermal sticking occurs exclusively at crystal steps due to a 1.5 eV difference between the activation barrier at terrace (∼1.9 eV) and step (∼0.4 eV) sites,^20,42−44^ illustrating the role step edges and defects can play in adsorption on single-crystal model systems.^45,46^ Molecular beam studies^19−21,23−25,47^ demonstrate that high impinging kinetic energies of N_2_ activate dissociation on terrace sites, with no dependence on surface temperature. The dissociation probability increases slowly with increasing N_2_ kinetic energy and plateaus at *s*_0_ ≈ 10^–2^ for kinetic energies much higher than the activation barrier;^19−21^ while molecular beams of N_2_ seeded in He and H_2_ carrier gases demonstrate how vibrational excitation of the impinging N_2_ increases the dissociation probability markedly,^19^ nonthermal plasmas have been used to populate vibrational and electronic excited states, thereby increasing reactivity of N_2_ with Ru-based catalysts.^48−50^ Additionally, measured isotope effects for N_2_ dissociation^24^ and hydrogenation^51^ of atomic nitrogen make N/Ru(0001) a suitable system for the study of nonadiabatic tunneling mechanisms.

STM studies,^52,53^ in conjunction with theoretical calculations,^1,42,43^ have helped elucidate the spatial properties, adsorbate–adsorbate interactions, and binding structures of adsorbed nitrogen atoms with atomic resolution. At low temperatures, molecular N_2_ adsorbs to Ru(0001) binding perpendicularly at on-top sites, and molecularly bound N_2_ desorbs from Ru(0001) at temperatures greater than 128 K^54^—ensuring that adsorbed molecular N_2_ will not be found in STM images within this study. STM visualization depicts adsorbed nitrogen atoms as triangular depressions 5 Å wide in topographical scans.^52,53^ N adsorbates (N_ad_) occupy the hcp 3-fold hollow site on terraces^52^ and bridge site on steps^43^ of the Ru(0001) surface. Interactions between N_ad_ are repulsive at nearest-neighbor and second-nearest-neighbor sites and slightly attractive at third-nearest-neighbor sites, resulting in an approximate pair potential of hard spheres that blocks the first- and second-nearest-neighbor sites in the low coverage regime at room temperature.^52^ Experimental^53^ and theoretical^1^ barriers to diffusion of 0.9 and 1.1 eV, respectively, have been observed for N_ad_ on the Ru(0001) surface. This allows adsorbate movement to be frozen out kinetically at moderate temperatures after the dissociation event occurs.

Molecular beam studies have investigated adsorption^19−21,23,24^ and inelastic scattering^25,47^ of N_2_ on Ru(0001); however, the techniques used heretofore are not directly suited to investigate energy dissipation during/after dissociative adsorption. Inelastic scattering provides information about the translational energy and quantum vibrational and rotational states of reflected molecules, but inelastic scattering notably looks at molecules that have been scattered from the surface and does not directly probe the reaction dynamics of molecules that adsorb to the surface. Temperature programmed desorption (TPD) provides initial sticking coefficients for various N_2_ beam energies, but each value only represents the ensemble average of a Boolean value—whether the molecule adsorbs or not—convoluting a multistep process (including energy loss in collision, trapped precursor states, diffusive/ballistic motion of atomic nitrogen, and energy loss to phonons and electronic friction) into one scalar value. Instead of measuring ensemble values, our study captures the results of individual dissociation events of a process that demonstrates an atypical sticking coefficient (*s*_0_ ≪ 1 at *E*_*N*_ ≫ V*).

The results in this paper represent the visualization of individual dissociation events resulting from nonequilibrium fluxes of energy- and angle-selected N_2_ impinging on Ru(0001) terrace sites. STM images from this study will serve as a benchmark for future computational models to provide insight into how energy is transferred in the highly activated dissociation of N_2_ on Ru(0001). This work provides a deeper understanding of N_2_ dissociation on ruthenium and in collaboration with theoretical exploration can contribute a more fundamental understanding of activated dissociative adsorption systems.

## Methods

The results reported in this paper were acquired utilizing a new UHV instrument that contains both a supersonic molecular beam and STM/AFM techniques. As reported in previous publications,^12,55−57^ the instrument is composed of a triply differentially pumped beamline, a surface characterization/preparation chamber that contains Auger electron spectroscopy (AES) and low-energy electron diffraction (LEED) capabilities, and a scanning probe microscope (SPM) chamber that holds the variable temperature SPM based on the ultrastable design of Shuheng Pan, built in collaboration with RHK. The custom-built PAN STM allows the Ru(0001) surface to be exposed to the supersonic molecular beam at variable polar angles of incidence with the ability to move the STM tip micrometers away from the area of interest to avoid blocking the molecular beam and then return the tip after the surface has been exposed to the molecular beam to reveal the morphology of the same nanoscopic area both before and after exposure to nonequilibrium fluxes of reactive gases.

Supersonic molecular beams were generated by the expansion of a 3% N_2_/97% He gas mixture through a 30 μm molybdenum pinhole at pressures from 20 to 100 psi and nozzle temperatures ranging from 300 to 1150 K (±5%). The translational kinetic energy of the molecular beam at each nozzle temperature was measured using time-of-flight (TOF), and values of 0.8 ± 0.3, 1.1 ± 0.4, and 1.3 ± 0.6 eV were found for nozzle temperatures of 730, 1000, and 1150 K, respectively. The uncertainty values in these energies represent the FWHM of each energy distribution. The molecular beam flux at the crystal for all beam conditions was on the order of 10^13^ N_2_ molecules cm^–2^ s^–1^. The kinetic energy values reported represent only translational kinetic energy, and the role of vibrational excitation on spatial distributions is not directly quantified in this study. A Boltzmann distribution with nozzle temperatures of 730, 1000, and 1150 K indicates, assuming, with no relaxation during expansion, populations of vibrationally excited impinging N_2_ molecules are 0.97%, 3.4%, and 5.5%, respectively.

All N_2_ molecular beam exposures onto the Ru(0001) surface were performed with the sample in the SPM chamber, which corresponds to a 4 mm diameter beam spot on the crystal. The sample was either held at room temperature or cryocooled with liquid nitrogen during imaging/exposures; the temperature of the sample was monitored using a cryostat thermocouple attached to the STM assembly. The surface temperature was held constant between exposure and imaging for each experiment. The surface plane could be oriented to achieve an incident polar angle from 0 to 45° during exposures to the N_2_ molecular beam.

Reactive evolution of the surface only arose due to exposure to the N_2_ molecular beam and not as a result of trace thermalized N_2_ reflected from the tip/chamber, which was confirmed by the fact that only areas of the surface with direct line of sight to the N_2_ beam were reacted.

The Ru(0001) crystals (Surface Preparation Laboratory, 99.99% purity) used in all experiments were cleaned in the characterization/preparation chamber (<1 × 10^–10^ Torr base pressure) by multiple sputter/anneal cycles, similar to those reported previously.^58,59^ The Ru(0001) surface was sputtered at room temperature using 0.5 keV Ar^+^ ions generated by a PHI 04-150 ion gun resulting in a current of 0.1–0.5 μA cm^–2^ on the sample; the sample was flash annealed by electron beam bombardment to 1500 K for 10 s after sputtering cycles. The temperature was monitored using a Mikron infrared pyrometer (ε = 0.35) during annealing. Hundreds of cleaning cycles were necessary to produce a clean and ordered Ru(0001) surface. An Omicron NGL 10 SPECTALEED with both LEED and AES capabilities was used to determine if the Ru(0001) surface was ordered and free of impurities.

**Figure 1:**
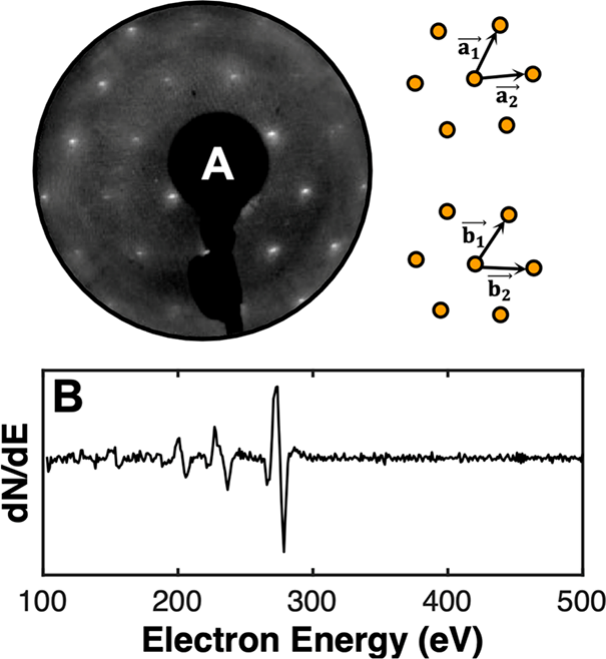
(A) A LEED pattern of the clean Ru(0001) surface demonstrating a sharp, hexagonal pattern with higher order diffraction peaks; a schematic of the reciprocal ( and ) and real ( and ) space lattice vectors is given, corresponding to the interpretation of the LEED pattern and STM images, respectively. (B) A differentiated AES spectrum (3 keV) of the clean Ru(0001) surface showing no contaminant peaks.

Initially, lower energy (0.5 keV) Ar^+^ sputtering cycles followed by longer anneal cycles (>1 min) at slightly lower temperatures (<1400 K) were used and produced a partially clean surface. Local areas of clean and ordered Ru(0001) surface were produced from these procedures, but subsequent STM imaging revealed the presence of Moiré patterns indicative of monolayer and bilayer graphene formation.^60^ Subsequent higher energy (3 keV) Ar^+^ sputtering cycles followed by shorter (5 s) and higher temperature (1500 K) annealing cycles produced a clean, ordered Ru(0001) surface, characterized by AES, LEED, and STM visualization in Figure 1. STM images were taken using etched or cut Pt_0.8_Ir_0.2_ tips.

## Results and Discussion

**Figure 2:**
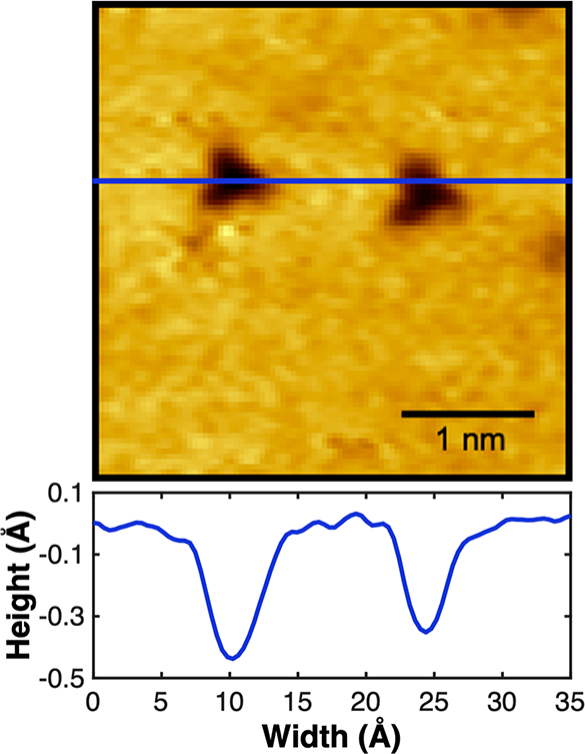
A representative STM image (−1.5 V, −250 pA) acquired at room temperature of two adsorbed nitrogen atoms, imaged as dark triangles. A line scan is shown to demonstrate the width and depth of the visualized nitrogen complexes. The observed N coverage is 0.01 ML.

Once a clean Ru(0001) surface was achieved and characterized, the surface was exposed to N_2_. As shown in Figure 2, adsorbed nitrogen atoms are imaged as triangular depressions approximately 5 Å wide and 0.5–1 Å deep in topographical scans, closely matching literature values;^52,53^ the 3-fold symmetry of the adsorbate suggests binding at either the fcc or hcp sites, with previous experimental and theoretical studies indicating binding at hcp sites is most favorable.^1,52,61^

**Figure 3:**
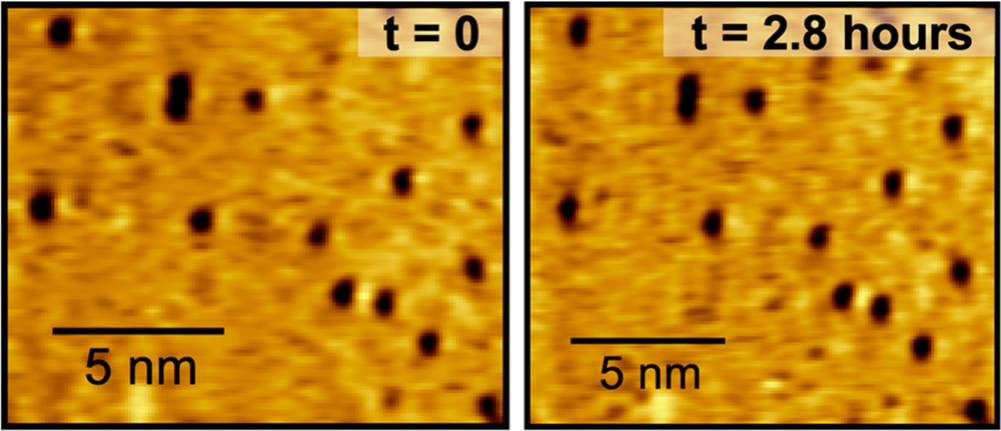
STM images (−1.5 V, −250 pA) of the same nanoscopic area were taken 2.8 h apart and acquired at room temperature. The N coverage is 0.004 ML.

STM imaging of the same nanoscopic area revealed no observable diffusion of the nitrogen atom adsorbates (Figure 3).^12^ Contrary to a previous STM study,^53^ these “diffusion” experiments were performed multiple times on the room temperature Ru(0001) surface, and no observable diffusion was observed over the course of hours. The results can be explained most likely by the use of a tunneling current in this study that is <100 times that used previously,^53^ thereby minimizing tip interactions with the adsorbates that can influence their movement upon the surface. To further elaborate that room temperature was sufficient to freeze diffusion and rule out small fluctuations in room temperature generating markedly different adsorbate distributions on the surface, the Ru(0001) surface was held at 262 K to probe the effect of temperature changes on adsorbate distributions. As expected, the lower surface temperature showed no effects on spatial distributions of adsorbates. Annealing above 1500 K provides an atomically clean surface for N_2_ molecular beam exposures, but coadsorbed species such as oxygen were observed after several hours of STM imaging from residual background dosing. O_ad_ was not observed to promote N_ad_ diffusion at this low coverage limit and is thus not expected to impact the spatial distributions of N_ad_ measured in this study. Notably, the lack of diffusion enables the N_2_ dissociation event to be directly investigated at room temperature by measuring the distance between and location of the resulting nitrogen atom adsorbates. STM images of atomic nitrogen adsorbate pairs thus provide direct insight into the energy transfer of the impinging N_2_ with the surface and the liberated energy during the dissociation event and do *not* show the effects of random thermal diffusion.

**Figure 4:**
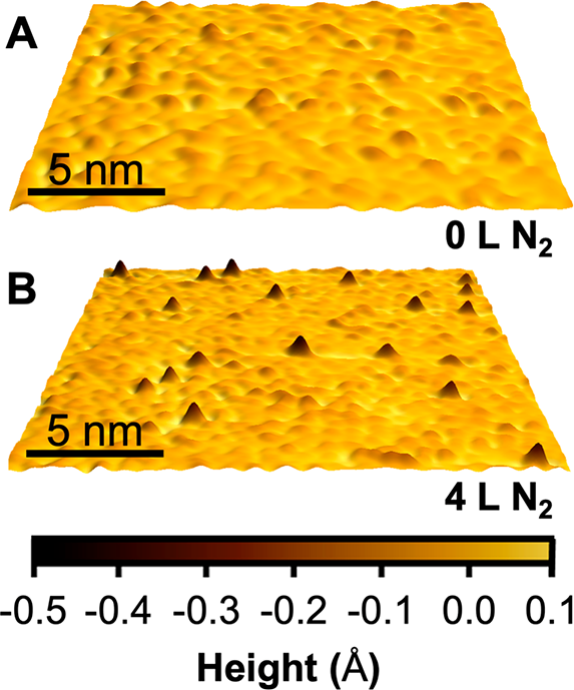
Representative room temperature STM images (−1.5 V, −250 pA) of a (A) clean Ru(0001) surface and (B) a Ru(0001) surface directly after exposure to 4 langmuirs of 1.1 eV N_2_ impinging 45° to the surface. The average total translational kinetic energy was 1.1 eV; parallel and perpendicular components of impinging kinetic energy are both 0.55 eV for this exposure. The N coverage is 0.003 ML in (B).

Shown in Figure 4 are representative STM images of the room temperature Ru(0001) surface before and directly after exposure to 4 langmuirs of 1.1 eV N_2_ impinging 45° to the surface normal, where 1 langmuir corresponds to one impinging N_2_ for every one Ru surface atom. In the low coverage regime, the reactivity of 1.1 eV N_2_ impinging normal to the Ru(0001) surface does not significantly differ from the reactivity of the same energy N_2_ impinging 45° with respect to the surface plane, suggesting that reactivity scales with total N_2_ energy, corroborating results from a previous study that showed molecular N_2_ adsorption on Ru(0001) is only weakly affected by incident angle when N_2_ translational kinetic energies are >0.4 eV.^62^ N_2_ molecules impinging normal and 45° to the surface with 1.1 eV translational kinetic energy were measured to have an approximately 1 in 10000 chance of sticking, which is in qualitative agreement with previous molecular beam studies.^19,20,47^

**Figure 5:**
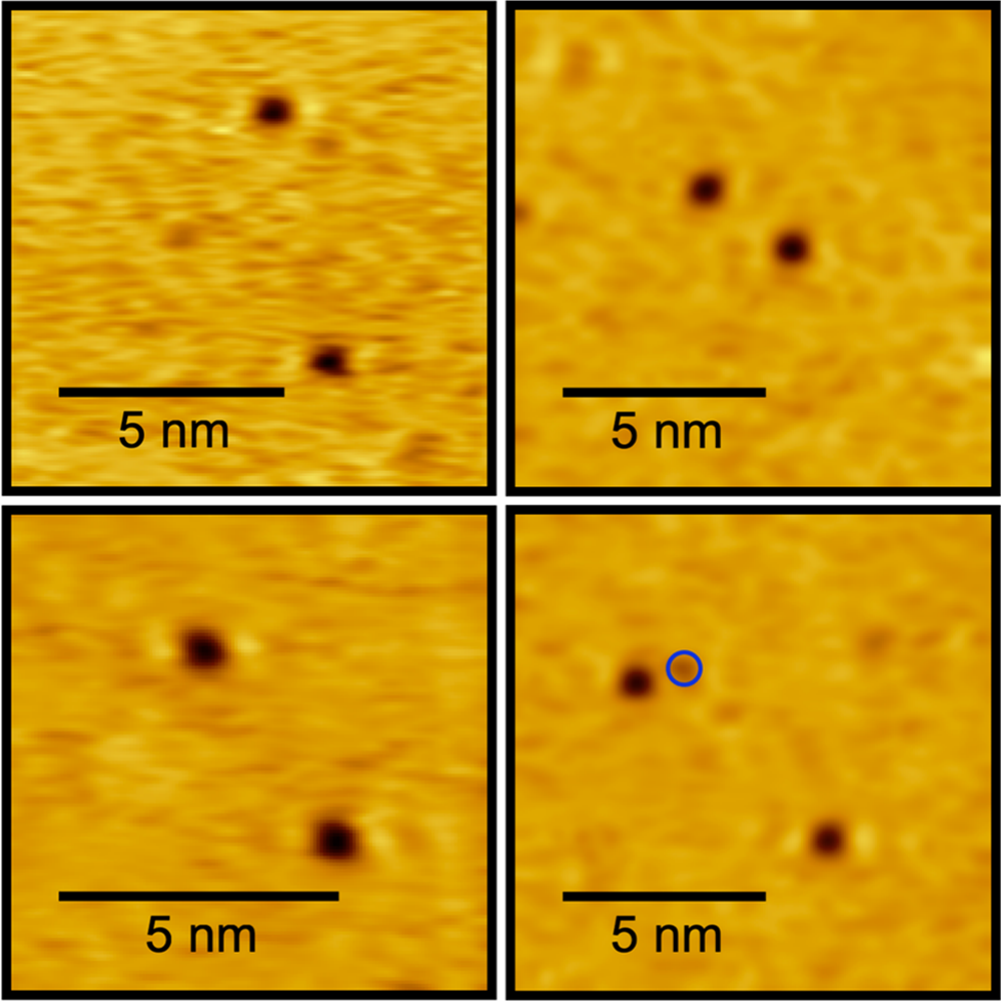
Representative STM images (−1.5 V, −250 pA) of nitrogen adatom pairs on a room temperature Ru(0001) surface after exposure to 0.6 langmuir of 1.1 eV N_2_ impinging normal to the surface. An oxygen adsorbate is circled in blue in the bottom right panel. The depth profile of oxygen adsorbates is less prominent than nitrogen adsorbates, making residual oxygen easily distinguishable from nitrogen. O coverage was noticed to increase over hours of STM imaging from trace background dosing.

Uncovering the pair distance(s) and the relative angle(s) between two adsorbates and the azimuthal direction of the incident molecular beam, and whether the impinging N_2_ kinetic energy and angle affect these values, is crucial to elucidating how the N_2_ interacts with the surface and what energy transfer mechanisms govern the reaction. Determination of the intrapair distances and relative angles of nitrogen pairs was accomplished by reacting at extremely low coverages (<0.08%) to reduce the total number of nitrogen atom pairs such that the identity of the nitrogen atom pairs could be uniquely assigned. Representative STM topography of the low coverage exposures is shown in Figure 5 for 1.1 eV N_2_ impinging normal to the Ru(0001) surface which clearly highlights *individual nitrogen pairs formed from singular dissociation events*. Misidentification of adsorbates pairs is avoided, as clean surfaces were always observed before reacting with N_2_ fluxes (as demonstrated in Figure 4), and the most prevalent contaminant observed on Ru(0001), oxygen, is easily distinguishable from nitrogen. The depth profile of oxygen adsorbates in STM images is less prominent than that of nitrogen using the selected tunneling conditions, as illustrated by a direct comparison of nitrogen adsorbates in Figure 5. STM images that revealed multiple dissociation events in the same nanoscopic area were not included in data analysis to avoid misidentification of nitrogen pairs.

**Figure 6:**
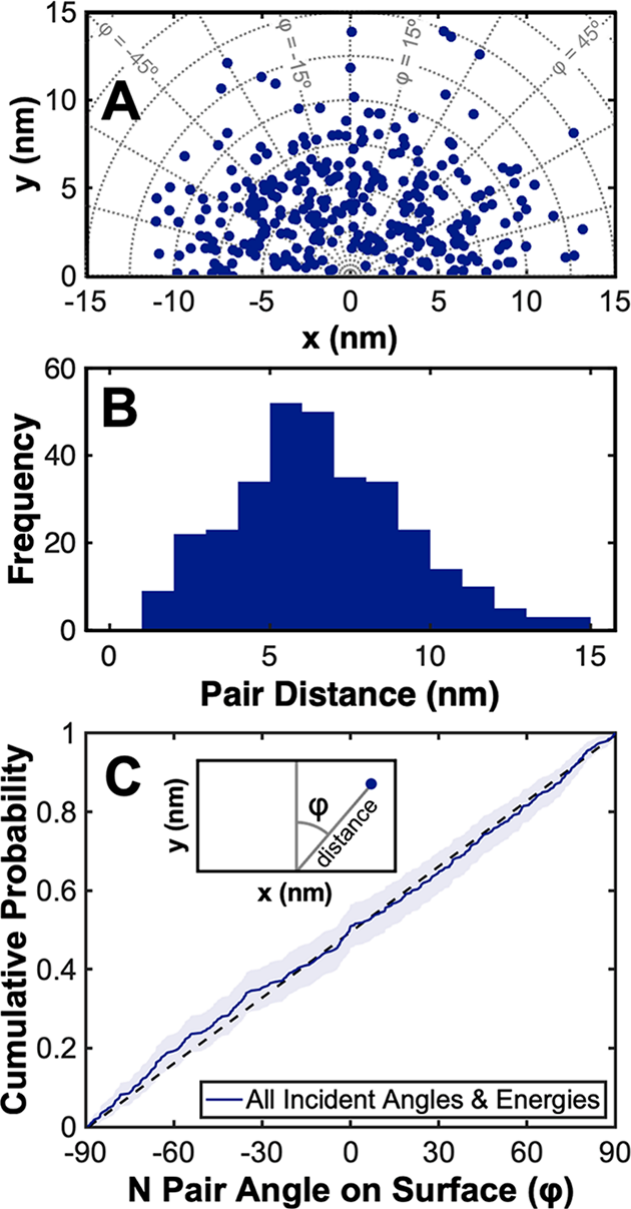
(A) A scatter plot shows the outcome of all nitrogen dissociation events observed in this study across all N_2_ impinging energies (total translational kinetic energies of 0.85, 1.1, and 1.3 eV) and impinging angles (45° and 90°). Each point in (A) represents a pair of nitrogen atoms from the same molecule in an STM image. (B) A histogram of distances between atoms from the same N_2_ molecule is shown. The Ru–Ru lattice constant is 2.7 Å, so 1 nm ≈ 3.7 Ru–Ru lattice constants. (C) A density function with a 95% confidence interval shows the distribution of azimuthal angles (φ) with respect to the incident azimuthal molecular beam direction for all dissociation events observed in (A). A straight line representing an isotropic distribution has been added to guide the eye. STM images used to generate these plots were acquired at either 300 or 262 K.

Extremely low coverage exposures of N_2_ on Ru(0001) were performed with varied translational kinetic energies and impinging angles. Polar coordinates (distance, φ) are used to describe the relative position of two nitrogen adsorbates from the same dissociated N_2_ molecule on the Ru(0001) lattice, where φ is the azimuthal angle between two adsorbates relative to the incident azimuthal direction of the molecular beam. Figure 6 shows the relative position of nitrogen adsorbate pairs from the same dissociated molecule across all reactant N_2_ translational kinetic energies and impinging angles used in this study.

A scatter plot (Figure 6A) demonstrates the relative location of nitrogen adsorbate pairs observed as a result of 318 individual dissociation events. The average intrapair distance for all observed dissociation events was 6.6 ± 2.8 nm with a sample size *N* = 318. The uncertainty of pair distances is reported as standard deviation throughout this paper. Intrapair spacing did not change significantly upon increasing the translational kinetic energy of N_2_ impinging normal to the surface from 0.85 eV (6.1 ± 2.1 nm, *N* = 56) to 1.3 eV (6.0 ± 2.4 nm, *N* = 126), indicating that there is not a strong coupling of impinging translational energy to intrapair distance for the energies explored herein. The distances measured across all molecular beam energies are extremely large considering that values are over 20 times larger than the lattice constant of ruthenium.

Comparisons to other dissociative chemisorption systems highlight the significance of these results. Background dosing of O_2_ on Pt(111),^63^ Al(111),^64^ Cu(110),^65^ Rh(110),^66^ TiO_2_(110),^67^ and RuO_2_(110)^68^ leaves oxygen atoms from the same dissociated O_2_ molecule within several lattice spacings on the surface, i.e., <1 nm apart. An early study of O_2_ on Al(111)^69^ indicated dissociative products may be spaced >80 Å apart, developing the notion of hot adatom motion, and more STM work followed showing “low transient mobility” after dissociation.^64^ Plasmon-induced dissociation of O_2_ on Ag(110) resulted in oxygen atoms within several lattice constants as well.^70^ The large intrapair distances observed for N_2_/Ru(0001) are not without precedent. Notable systems for far-ranged transient motion following dissociative chemisorption include O_2_ on Ag(001),^71^ in which intrapair distances were found to be either 2 or 4 nm, and Cl_2_ on TiO_2_(110),^72^ which demonstrates an average intrapair distance of 2.6 nm. A “cannonball mechanism” was proposed for Cl_2_/TiO_2_ in which one atom is ballistically propelled from the surface, thereby overcoming surface corrugation to explain the large distances observed; this ballistic motion that avoids corrugation in the potential energy surface could similarly explain the large intrapair distances observed for N_2_/Ru(0001) despite large barriers to diffusion and provides direction for future computational work. However, hundreds of identified N_ad_ pairs identified in this study and a lack of lone N atoms discourage promoting an abstractive mechanism where one atom desorbs entirely from the surface. STM tip-induced dissociation of small molecules on single crystalline surfaces has also yielded products at nonadjacent binding sites with products from CH_2_I_2_/Cu(110),^73^*m*-iodopyridine/Cu(110),^74^ and O_2_/Ag(110)^75^ separated by several lattice constants and F and CF_2_ up to 5.3 nm apart after CF_3_ dissociates on Cu(110).^76^ These previously studied model systems demonstrate that relatively large intrapair distances do occur for specific systems.

Note that such large distances observed provide important insight into elucidating the operative energy dissipation channels for a given system. Adiabatic processes will contribute dissipation primarily via phonon channels, while nonadiabatic processes on excited states and including ehps when appropriate can account for interesting dynamics. Preliminary results from *ab initio* density functional theory (DFT) calculations by Prof. Hua Guo’s research group are getting underway. Mechanistically, how these large intrapair distances are achieved despite a large barrier (0.9–1.1 eV)^1,53^ to diffusion poses interesting questions for future computational modeling. Does a purely adiabatic picture describe the N_2_/Ru(0001) system in which a nonthermalized hot adatom diffuses on the surface until phonons dissipate energy from the adsorbate into the bulk? Or do the dynamics sample excited neutral and ionic potential energy surfaces leading to changed overall dynamics? Beyond identifying the average distance between adsorbates, STM images provide a rich spatial understanding of the N_2_/Ru(0001) system.

STM images showed that nitrogen adsorbate pairs were not observed to preferentially align themselves in any direction on the Ru(0001) lattice. We observe that points are isotopically distributed between −90° and 90° in the scatter plot (Figure 6 A), and the linear trend in the cumulative density plot in Figure 6C emphatically shows this. An isotropic distribution in the orientation of nitrogen atom pairs (φ) provides insight into the mechanism for nitrogen dissociation on the surface by indicating the long-range motion of atomic species is not constrained to the orientation of the molecule’s dissociative transition state or any particular direction on the Ru(0001) lattice.

**Figure 7:**
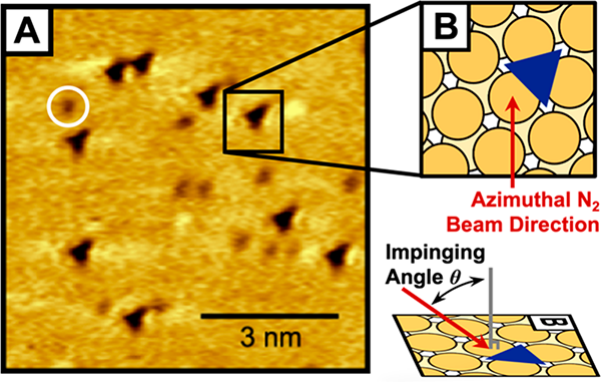
(A) STM image (−1.5 V, −250 pA) acquired at room temperature of the Ru(0001) surface with submonolayer coverages of nitrogen (dark triangles) and residual oxygen (example circled in white) adsorbates. O coverage was noticed to increase over hours of STM imaging from trace background dosing. (B) Schematic on the right highlights the orientation of the Ru(0001) lattice with respect to the azimuthal direction of the impinging N_2_ beam. The hcp binding site orientation on terraces dictates the orientation of the triangular motif of adsorbates in STM images, thereby unambiguously providing the orientation of the underlying Ru(0001) lattice.^52,77^ The N coverage is 0.1 ML.

Analysis thus far has focused on the aggregated results of all impinging N_2_ angles and energies found in Figure 6; however, STM imaging following reactions with variable reactant energies and impinging angles provides valuable information for elucidating the reaction mechanism. A schematic showing the impinging angle and the azimuthal direction of the incident molecular beam on the surface is given in Figure 7, which illustrates the orientation of the incident N_2_ molecular beam with respect to the Ru(0001) lattice—confirmed by STM imaging of the nitrogen adsorbates^52,77^—critical to determining correlations between the incident angle of N_2_ and the resulting nitrogen pair spacing, relative angle, and binding sites.

**Figure 8:**
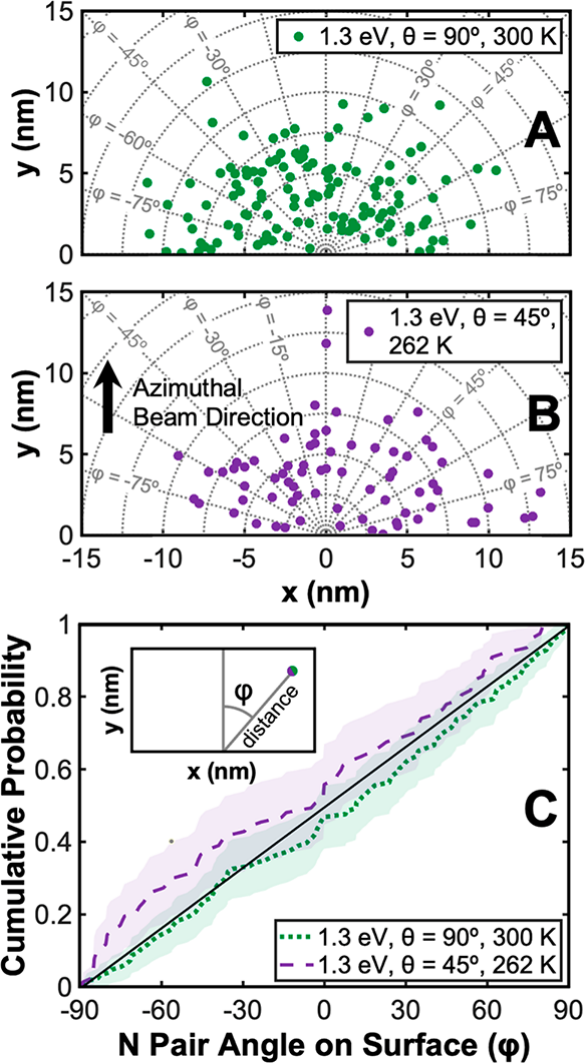
Scatter plots show the outcome of 1.3 eV N_2_ impinging (A) normal to a 300 K Ru(0001) surface and (B) 45° to a 262 K Ru(0001) surface. Each point in (A) and (B) represents a pair of nitrogen atoms from the same molecule in an STM image. The average total translational kinetic energy was 1.3 eV for both experiments; parallel and perpendicular components of impinging kinetic energy are both 0.65 eV for the 45° exposure. (C) Density functions with 95% confidence intervals show the distribution of azimuthal angles (φ) with respect to the incident azimuthal molecular beam direction from points in (A) and (B). A straight line representing an isotropic distribution has been added to guide the eye. STM images used to generate these plots were acquired at 300 and 262 K for N_2_ impinging 90° and 45°, respectively.

The distribution of adatom pairs following exposure to 1.3 eV N_2_ impinging 90° and 45° to the Ru(0001) surface is shown in Figure 8. No significant differences are observed between the results of these reactions as shown through several metrics.

The scatter plots shown in Figure 8 demonstrate an isotropic distribution of points with respect to the azimuthal angle φ—a trend emphasized by the fact that a completely isotropic angular distribution, given as a straight line for reference in Figure 8C, is within the 95% confidence interval for results from both normal and 45° impinging fluxes. This result provides important mechanistic information, as it shows that there is *not* an obvious memory function in which the relative angle (φ) between nitrogen adsorbates is affected by the non-normal component of momentum of incident N_2_ impinging at 45° to the surface. Similarly, pairs that were more aligned with the azimuthal incident beam direction (i.e., φ ≈ 0°) in Figure 8B were not observed to have larger intrapair distances than those not aligned with the molecular beam, indicating that there is not a strong ballistic memory function in which molecular momentum is coupled into the motion of one adsorbate along the direction of the impinging molecule.

**Figure 9:**
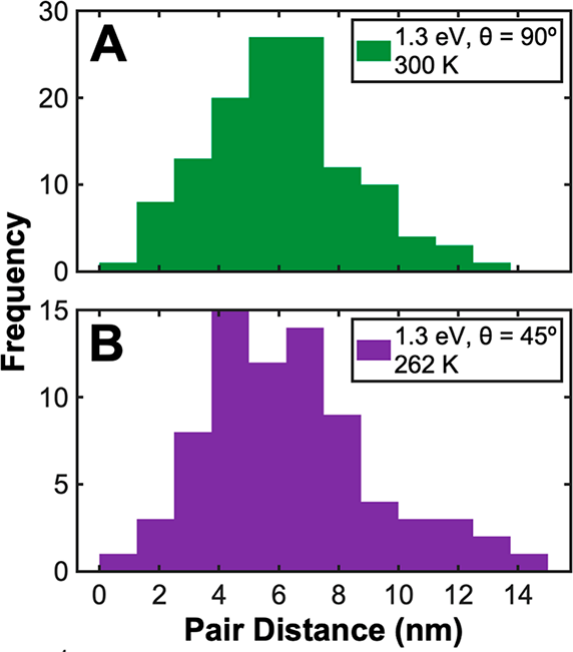
Histogram data showing measured adatom pair distances resulting from N_2_ with 1.3 eV translational energy impinging (A) normal to a 300 K surface and (B) 45° to a 262 K Ru(0001) surface. STM images used to generate these plots were acquired at 300 and 262 K, respectively. The average total translational kinetic energy was 1.3 eV for both experiments; parallel and perpendicular components of impinging kinetic energy are both 0.65 eV for the 45° exposure. The number of pairs for each histogram was *n*_A_ = 126 and *n*_B_ = 75. The Ru–Ru lattice constant is 2.7 Å, so 1 nm ≈ 3.7 Ru–Ru lattice constants.

Analogously, Figure 9 provides markedly similar histograms of intrapair distances resulting from the dissociation of N_2_ with 1.3 eV total translational kinetic energy impinging at both 45° and 90° to the surface, and average intrapair distances resulting from 1.3 eV impinging normal (6.0 ± 2.4 nm, *N* = 126) and impinging at 45° (6.4 ± 2.9 nm, *N* = 75) are similar as well. This result indicates that under these conditions, momentum along the azimuthal direction of the impinging N_2_ is not strongly coupled into motion of one N_ad_ along the Ru(0001) surface following dissociation. Results summarized in Figures 8 and 9 have thus indicated that N_2_ impinging at non-normal angles does not result in an obvious memory function for adsorbate spacings or angular distributions. These results are made more compelling by the fact that the angled N_2_ exposure (1.3 eV, 45°) was performed below room temperature (262 K) so that any potential random isotropic motion following dissociation would be frozen out.

Lowering the temperature of the Ru(0001) surface to 262 K during exposure to 1.3 eV N_2_ impinging 45° to the surface normal did not demonstrate a markedly different trend in adsorbate distributions on the surface from other room temperature runs (Figures 8 and 9), indicating that thermal diffusion does not play an outsized role in the relatively large pair distances observed—supplementing the conclusions drawn from “diffusion” experiments where no diffusion of nitrogen adsorbates on room temperature Ru(0001) was noticed over the course of hours as represented in Figure 3. It could be hypothesized that lowering the surface temperature to 262 K may *decrease* the intrapair spacing by freezing out diffusion of atomic species and changing the impinging angle of reactant molecules from 90° to 45° may *increase* the average intrapair distance by a coupling of the non-normal component of momentum to the motion of atomic nitrogen. These two hypothetical situations could potentially have canceling effects; however, reacting a room temperature Ru(0001) surface with 1.1 eV N_2_ impinging at 45° did not produce markedly different results from 1.1 eV impinging at 90° to the surface—in either the spacing or the relative angle of the paired nitrogen atoms. (We refer to the 1.1 eV N_2_ experiments anecdotally as they are composed of smaller data sets, results from which are included in Figure 6 and the overall intrapair distance of 6.6 ± 2.8 nm.) We surmise that neither incident angle nor a change in surface temperature (300 K vs 262 K) affect the distribution of adsorbates on the surface.

The results of this paper represent deep insight into the fundamental dynamics of N_2_ activated dissociative adsorption on Ru(0001). STM visualization of individual dissociation events identifies the site and location of the resulting nitrogen adsorbates with angstrom-level precision. The spacing of the adsorbates correlates to the energy transfer mechanisms involved in the dissociation process and will help uncover the importance of nonadiabatic excitation of ehps in these reactive events. Paired with theoretical insight from ongoing collaborations, these previously inaccessible experimental results will help to answer many of the unsolved questions about this unusual activated dissociative adsorption system.

## Conclusions

We present STM images depicting the products of individual dissociation events following nonequilibrium fluxes of energy- and angle-selected N_2_ from a supersonic molecular beam impinging on a Ru(0001) single crystal. The insights gathered herein are only possible due to the prescient pairing of a supersonic molecular beam of highly energetic molecules and the angstrom-level visualization of an *in situ* in-line STM to provide a rich spatial understanding of this highly activated dissociative adsorption system. Our work uniquely characterizes single adsorption events to capture the reactive dynamics of this industrially relevant process. We present distributions of nitrogen adsorbate pairs resulting from highly energetic N_2_ impinging onto Ru(0001), resulting in an average spacing of 6.6 ± 2.8 nm between previously bonded nitrogen atoms. These distances are more than 20 times the lattice constant of Ru(0001) and pose a novel opportunity for theoretical formulations to investigate the importance of electronic friction, adiabatic phonon coupling, multielectronic surface dynamics, and ballistic motion in energy dissipation following activated dissociation events. When discussing the effect of N_2_ impinging energy and angle on the reactivity in a low coverage limit, our preliminary results suggest there is not an obvious memory function for incident momenta/kinetic energy of impinging N_2_ on the distance between resulting nitrogen adsorbates. Additionally, the non-normal component of impinging N_2_ momentum does not impact spatial distributions on the surface. Our results provide direct experimental insight into the energy transfer mechanisms and detailed aspects of the dissociation process and help address important and unresolved questions surrounding the fundamentally and technologically important activated dissociative adsorption process of N_2_ dissociation on Ru(0001).
